# Overactivation of Akt Contributes to MEK Inhibitor Primary and Acquired Resistance in Colorectal Cancer Cells

**DOI:** 10.3390/cancers11121866

**Published:** 2019-11-25

**Authors:** Masanobu Tsubaki, Tomoya Takeda, Masaki Noguchi, Minami Jinushi, Shiori Seki, Yuusuke Morii, Kazunori Shimomura, Motohiro Imano, Takao Satou, Shozo Nishida

**Affiliations:** 1 Division of Pharmacotherapy, Kindai University Faculty of Pharmacy, Kowakae, Higashi-Osaka 577-8502, Japan; tsubaki@phar.kindai.ac.jp (M.T.); takeda@phar.kindai.ac.jp (T.T.); noguchi_kindai_phar@yahoo.co.jp (M.N.); jinushi_kindai@yahoo.co.jp (M.J.); seki_kindai@yahoo.co.jp (S.S.); morii_kindai@yahoo.co.jp (Y.M.); 2 Department of Phamacy, Municipal Ikeda Hospital, Ikeda, Osaka 563-8510, Japan; shimomura_ikeda@yahoo.co.jp; 3 Department of Surgery, Kindai University Faculty of Medicine, Osakasayama, Osaka 589-0014, Japan; imano@med.kindai.ac.jp; 4 Department of Pathology, Kindai University Faculty of Medicine, Osakasayama, Osaka 589-0014, Japan.; takaosat@med.kindai.ac.jp

**Keywords:** MEK inhibitor, KRAS, BRAF, PIK3CA, colorectal cancer, cell culture, Akt

## Abstract

RAS and BRAF-mutated colorectal cancers are associated with resistance to chemotherapy and poor prognosis, highlighting the need for new therapeutic strategies. Although these cancers sometimes respond to mitogen activated protein kinase kinase (MEK) inhibitor treatment, they often acquire resistance via mechanisms, which are poorly understood. Here, we investigated the mechanism of MEK inhibitor resistance in primary- and acquired-resistant cells. Cell viability was examined using the trypan blue dye exclusion assay. Protein expression was analyzed by western blotting. Somatic mutations in colorectal cancer cells were investigated using the polymerase chain reaction array. PD0325901 and trametinib induced cell death in LoVo and Colo-205 cells but not in DLD-1 and HT-29 cells, which have a PIK3CA mutation constitutively activating Akt and NF-κB. Treatment with PD0325901 and trametinib suppressed ERK1/2 activation in all four cell lines but only induced Akt and NF-κB activation in DLD-1 and HT-29 cells. Inhibition of Akt but not NF-κB, overcame MEK inhibitor resistance in DLD-1 and HT-29 cells. Acquired-resistant LoVo/PR, Colo-205/PR and LoVo/TR cells have constitutively active Akt due to a M1043V mutation in the kinase activation loop of PIK3CA and Akt inhibitor resensitized these cells to MEK inhibitor. These results demonstrate that the overactivation of Akt plays a critical role in MEK inhibitor primary and acquired resistance and implicate combined Akt/MEK inhibition as a potentially useful treatment for RAS/BRAF-mutated colorectal cancer.

## 1. Introduction

Colorectal cancer is the third most common cancer worldwide and the most common cancer in Japan [1]. Although first-line therapy is surgical resection, patients with unresectable cancer and relapse generally undergo chemotherapy (5-fluorourasil, levofolinate and oxaliplatin/irinotecan combinations [FOLFOX/FOLFIRI regimen] or capecitabine and oxaliplatin combinations [XELOX regimen]). In addition, patients with KRAS-wild type colorectal cancer are treated with the epidermal growth factor receptor (EGFR) antibody cetuximab or panituzumab in combination with chemotherapy [2]. However, these chemotherapy and anti-EGFR combination treatments become less effective in patients with tumors harboring mutations [3,4,5].

Primary colorectal cancers frequently harbor somatic mutation of KRAS (~40%), BRAF (~10–15%) and PIK3CA (~10–20%) [6,7,8,9]. These mutations induce activation of the Raf/mitogen activated protein kinase kinase (MEK)/extracellular signal regulated kinase (ERK) and phosphoinositol-3 kinase (PI3K)/Akt pathways which contribute to resistance to chemotherapy and anti-EGFR treatments [3,4,5]. In addition, patients with colorectal cancer harboring KRAS, BRAF or PIK3CA mutations have poor prognosis compared to patients without these mutations [3,4,5]. Several studies have indicated that MEK inhibitors can suppress the proliferation of colorectal cancer cells harboring KRAS or BRAF mutations in vitro and in vivo [10,11,12]. In addition, combining MEK and PI3K inhibitors induced significant cell death in several human cancer cell types, including colorectal, lung, breast and pancreatic cancer [13,14,15,16,17]. However, the oral MEK inhibitor RO4987655 (CH4987655) was not effective against KRAS-mutated colorectal cancer in a phase I study [18]. A combination of the MEK inhibitor selumetinib and cetuximab also displayed minimal antitumor activity in KRAS mutant colorectal cancer [19]. In addition, a combination of the MEK inhibitor trametinib and panituzumab gave a response rate of 0% in BRAF mutant colorectal cancer [20]. Moreover, colorectal cancer cells often develop resistance to MEK inhibitors [21,22], though the specific mechanisms underlying this are not well understood.

In this study, we investigate the mechanisms of MEK inhibitor primary and acquired resistance and determine whether other inhibitors, such as Akt and NF-κB inhibitor, can be used to overcome MEK inhibitor primary and acquired resistance in human colorectal cancer cells harboring KRAS, BRAF and PIK3CA mutation.

## 2. Results

### 2.1. Sensitivity of Colorectal Cancer Cells to Trametinib and PD0325901

**Figure 1:**
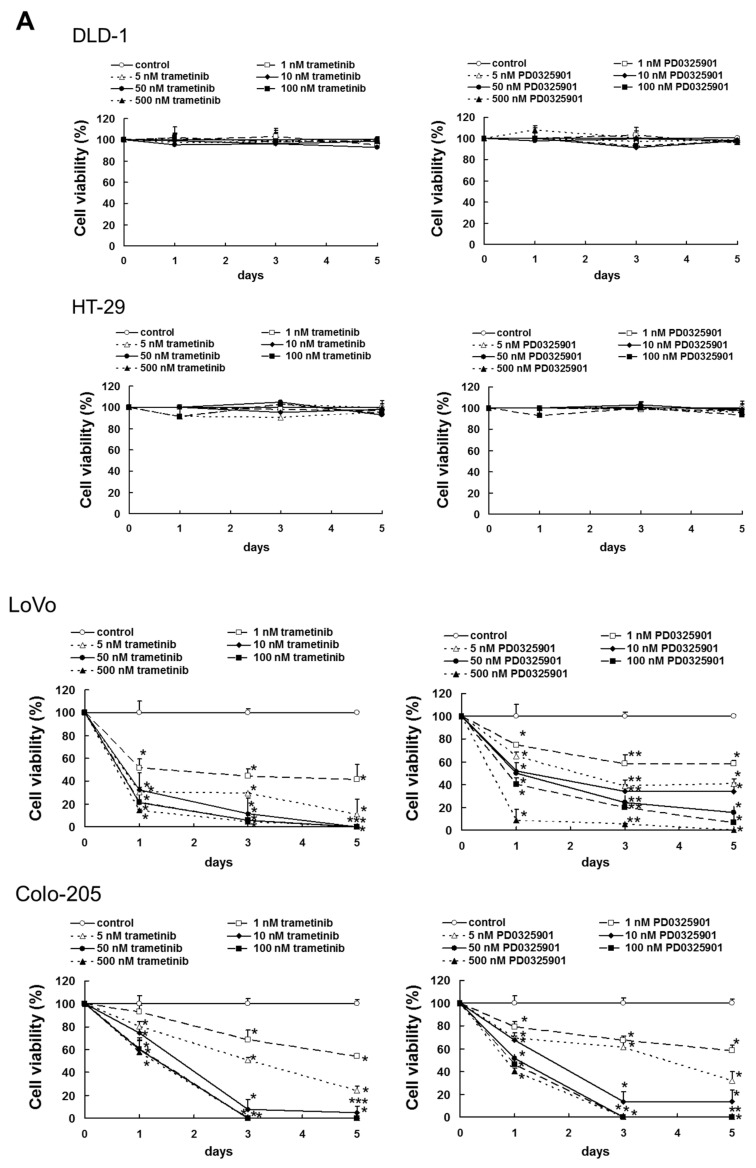

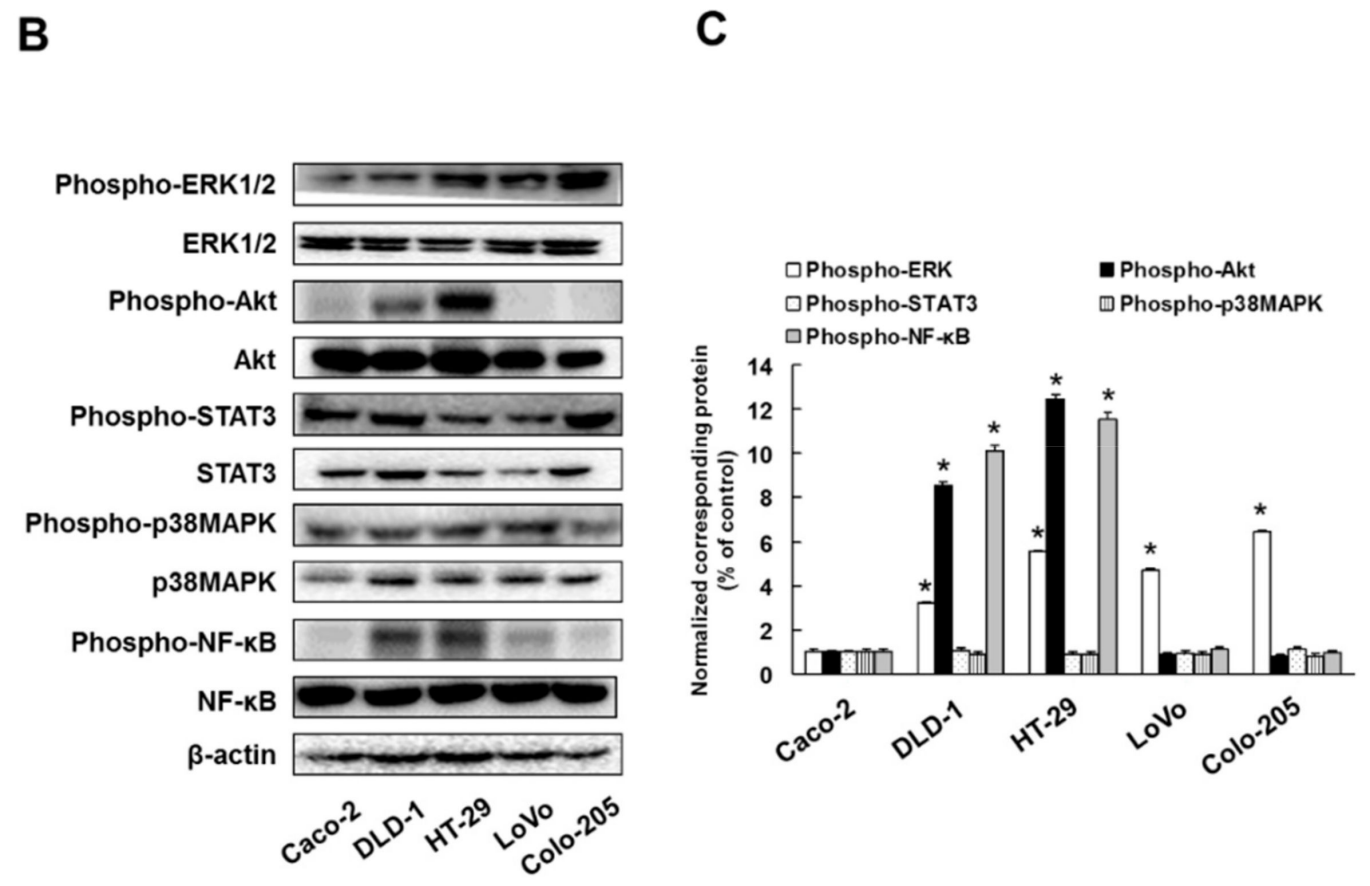
Effect of mitogen activated protein kinase kinase (MEK) inhibitor on human colorectal cancer cell viability. (**A**) Cell viability of trametinib- and PD0325901-treated DLD-1, HT-29, LoVo and Colo-205 cells as measured by the trypan blue dye assay. These cells were administrated with indicated concentrations of trametinib and PD0325901 for 1, 3 or 5 days. The results showed the 5 independent experiments. * *p* < 0.05, ** *p* < 0.01 vs. controls (Cell viability on DLD-1 and HT-29 cells were analyzed by Shapiro-Wilk test and one-way analysis of variance (ANOVA) with Dunnett’s test. Cell viability on LoVo and Colo-205 cells were analyzed by Shapiro-Wilk test and Kruskal-Wallis test followed by Steel test.). (**B**) Cell lysates were examined by western blotting assay using indicated antibodies. (**C**) Quantification of phosphorylated protein expression, normalized corresponding protein, respectively. The results showed the 5 independent experiments. * *p* < 0.01 vs. controls (Shapiro-Wilk test and ANOVA with Dunnet’s test).

We first examined the mutation status of the KRAS, PIK3CA and BRAF genes in human colorectal cancer cell lines. We confirmed that DLD-1 cells harbor hyperactivating mutations in KRAS (G13D) and PIK3CA (E545K), LoVo cells harbor a KRAS mutation (G13D) and HT-29 and Colo-205 cells harbor a hyperactivating BRAF mutation (V600E) (Supplementary Materials, Figures S1–S3). In addition, a previous report demonstrated that HT-29 cells have a hyperactivating mutation in PIK3CA (P449T) [23]. Next, we investigated the cytotoxic effects of trametinib (1–500 nM) and PD0325901 (1–500 nM) on DLD-1, HT-29, LoVo and Colo-205 cells. Whereas trametinib and PD0325901 did not induce cell death in DLD-1 and HT-29 cells, both drugs significantly induced cell death in LoVo (trametinib; *p* < 0.02 on day 1, *p* < 0.02 on day 3, *p* < 0.02 on day 5, PD0325901; *p* < 0.02 on day 1, *p* < 0.01 on day 3, *p* < 0.02 on day 5) and Colo-205 cells (trametinib; *p* < 0.02 on day 1, *p* < 0.02 on day 3, *p* < 0.02 on day 5, PD0325901; *p* < 0.02 on day 1, *p* < 0.02 on day 3, *p* < 0.02 on day 5) in concentration- and time-dependent manners (Figure 1A and Supplementary Materials, Figure S4).

To examine the activation status of molecules downstream of the KRAS, PIK3CA and BRAF signaling pathways, we assessed the phosphorylation levels of ERK1/2, Akt, signal transducer and activator of transcription 3 (STAT3), p38 mitogen-activated protein kinase (p38MAPK) and nuclear factor-kappa B (NF-κB) in DLD-1, HT-29, LoVo and Colo-205 cells. As a negative control we used Caco-2 cells, which contain no mutations in KRAS, PIK3CA or BRAF. The ERK1/2 phosphorylation level was higher in DLD-1 (*p* < 0.01), HT-29 (*p* < 0.01), LoVo (*p* < 0.01) and Colo-205 (*p* < 0.01) cells than in Caco-2 cells. In addition, Akt and NF-κB phosphorylation levels were elevated in DLD-1 (*p* < 0.01) and HT-29 (*p* < 0.01) cells but there is no difference between LoVo or Colo-205 cells and Caco-2 cells. However, the levels of phosphorylated STAT3 and p38MAPK did not differ between any of the cell lines (Figure 1B,C).

### 2.2. The Effect of Trametinib and PD0325901 on ERK1/2, Akt or NF-κB Activation in Colorectal Cancer Cells

**Figure 2:**
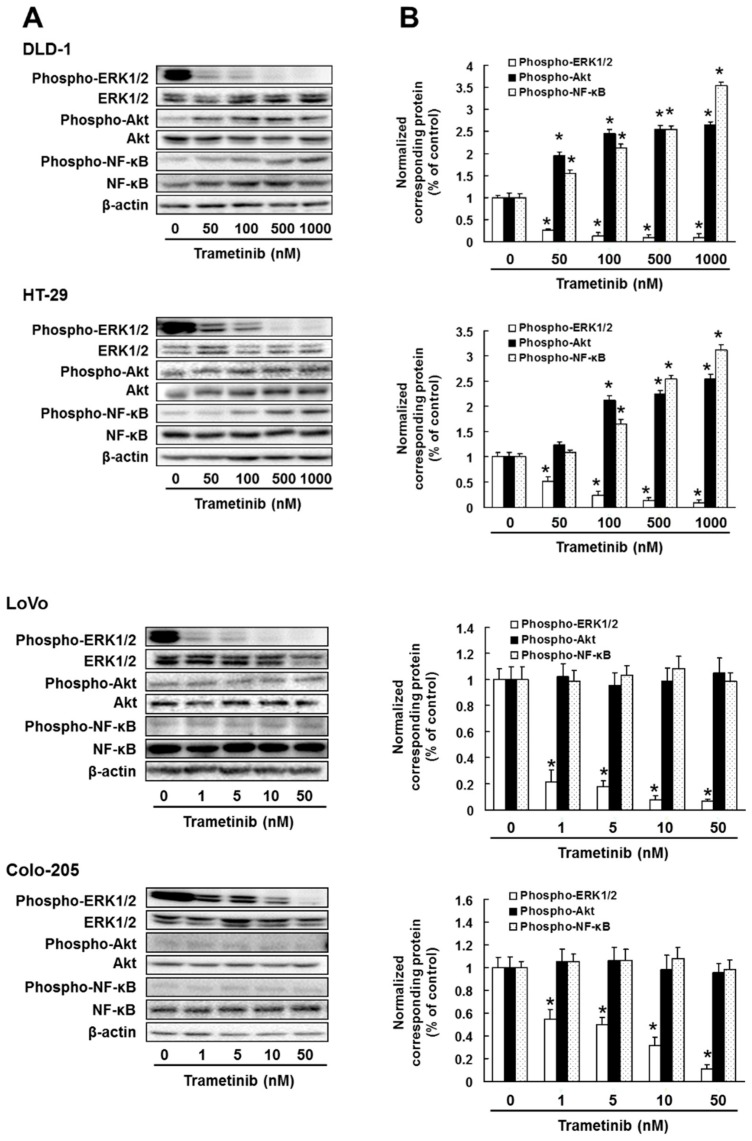

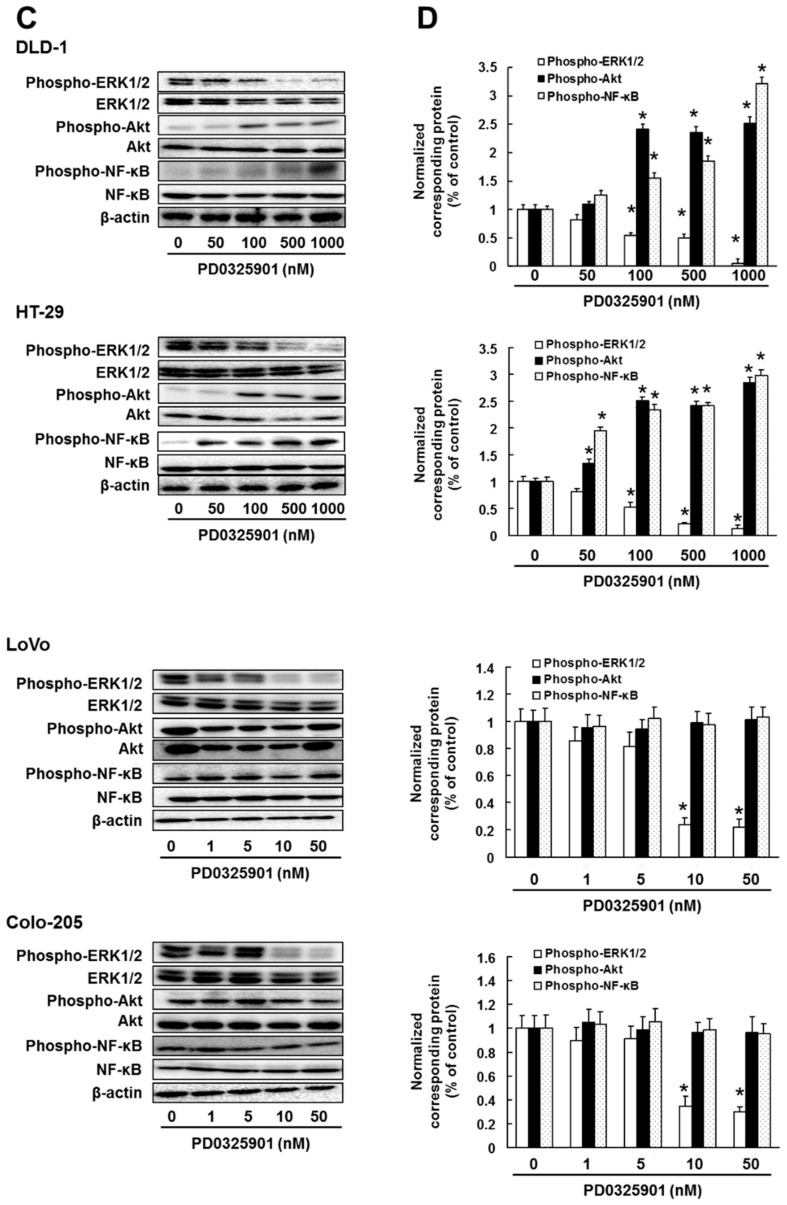
Effect of MEK inhibitor on ERK1/2, Akt and NF-κB activation. Cells were administrated with (**A**,**B**) trametinib or (**C**,**D**) PD0325901 for 3 days. Control cells (0 μM) were administrated with 0.5% dimethyl sulfoxide (DMSO) for 3 days. (**A**,**C**) Cell lysates were examined by western blotting assay using indicated antibodies. (**B**,**D**) Quantification of phosphorylated protein expression, normalized corresponding protein, respectively. The results showed the 5 independent experiments. * *p* < 0.01 vs. controls (Shapiro-Wilk test and ANOVA with Dunnet’s test).

**Figure 3:**
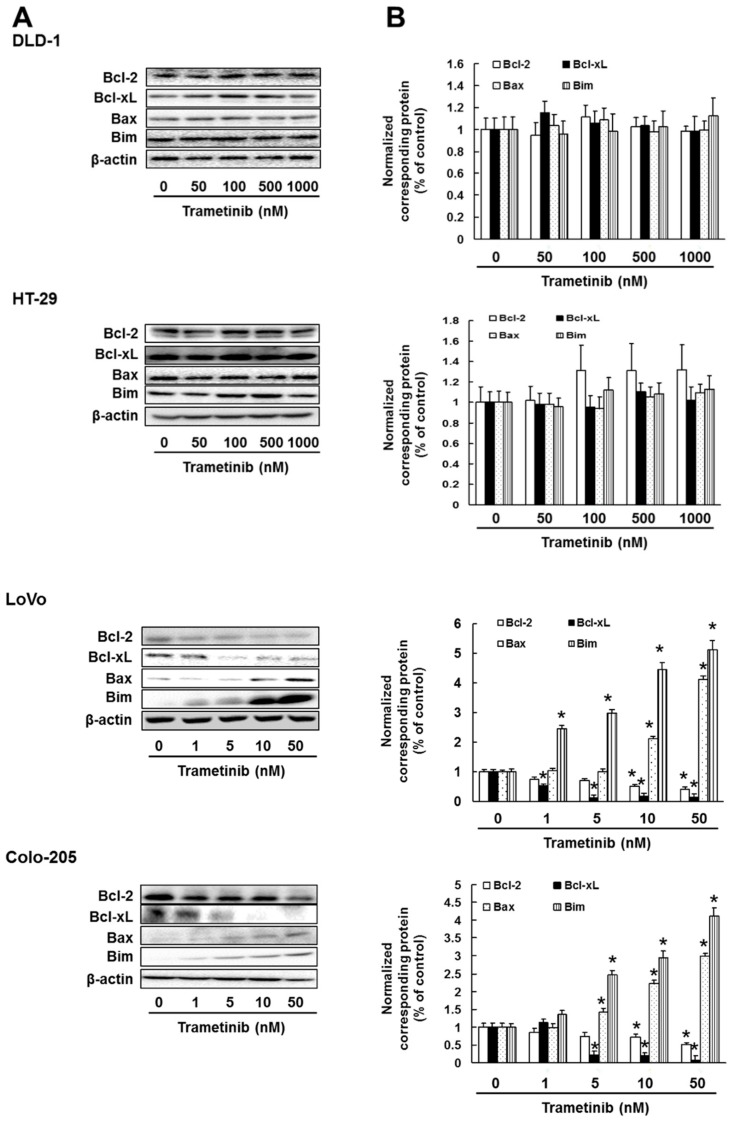
Effect of trametinib on Bcl-2, Bcl-xL, Bax and Bim expression. Cells were administrated with trametinib for 3 days. Control cells (0 μM) were administrated with 0.5% DMSO for 3 days. (**A**) Cell lysates were examined by western blotting assay using indicated antibodies. (**B**) Quantification of protein expression, normalized corresponding protein, respectively. The results showed the 5 independent experiments. * *p* < 0.01 vs. controls (Shapiro-Wilk test and ANOVA with Dunnet’s test).

Next, we investigated the effect of trametinib and PD0325901 on ERK1/2, Akt or NF-κB activation in DLD-1, HT-29, LoVo and Colo-205 cells. Treatment with trametinib and PD0325901 suppressed ERK1/2 phosphorylation in all cell lines in concentration-dependent manner (DLD-1: trametinib; *p* < 0.01, PD0325901; *p* < 0.01, HT-29: trametinib; *p* < 0.01, PD0325901, *p* < 0.01, LoVo: trametinib; *p* < 0.01, PD0325901; *p* < 0.01, Colo-205: trametinib; *p* < 0.01, PD0325901; *p* < 0.01). In addition, concentrations of trametinib and PD0325901 that significantly suppressed ERK1/2 activation also enhanced Akt and NF-κB phosphorylation in DLD-1 (trametinib; *p* < 0.01, PD0325901; *p* < 0.01) and HT-29 (trametinib; *p* < 0.01, PD0325901; *p* < 0.01) cells but not in LoVo and Colo-205 cells (Figure 2A–D). Moreover, concentrations of trametinib that significantly inhibited ERK1/2 activation suppressed expression of the survival factors B-cell lymphoma 2 (Bcl-2) and Bcl-2-like protein 1 (Bcl-xL) and enhanced expression of the pro-apoptotic factors Bcl-2 associated X protein (Bax) and Bcl-2 interacting mediator of cell death (Bim) in LoVo (Bcl-2; *p* < 0.01, Bcl-xL; *p* < 0.01, Bax; *p* < 0.01, Bim; *p* < 0.01) and Colo-205 (Bcl-2; *p* < 0.01, Bcl-xL; *p* < 0.01, Bax; *p* < 0.01, Bim; *p* < 0.01) cells in a concentration-dependent manner but did not affect the expression of any of these factors in DLD-1 and HT-29 cells (Figure 3).

### 2.3. Effect of Combined Treatment with MEK Inhibitors and Akt Inhibitor Perifosine or the NF-κB Inhibitor Dimethyl Fumarate (DMF), on DLD-1 and HT-29 Cell Viability

**Figure 4:**
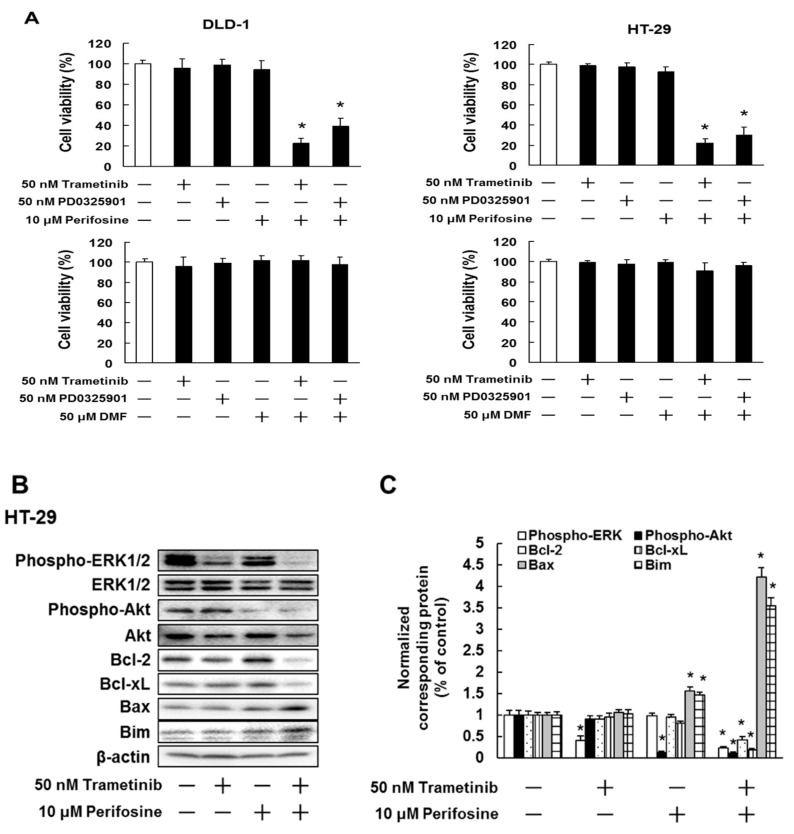
Akt inhibitor but not NF-κB inhibitor, overcomes MEK inhibitor primary resistance. (**A**) DLD-1 and HT-29 cells were administrated with the indicated concentrations of trametinib, PD0325901, perifosine or dimethyl fumarate (DMF). After incubation for 72 h, cell viability was analyzed by trypan blue staining. The results showed 5 independent experiments. * *p* < 0.01 vs. untreated cells as assessed with Shapiro-Wilk test and ANOVA with Dunnet’s test. (**B**,**C**) HT-29 cells were administrated with trametinib or perifosine for 3 days. (**B**) Cell lysates were examined by western blotting assay using indicated antibodies. (**C**) Quantification of protein expression, normalized corresponding protein, respectively. The results showed the 5 independent experiments. * *p* < 0.01 vs. controls (Shapiro-Wilk test and ANOVA with Dunnet’s test).

Given our results suggesting that the activation of Akt and NF-κB may be involved in MEK inhibitor primary resistance in colorectal cancer cells, we investigated whether perifosine or dimethyl fumarate (DMF) could overcome MEK inhibitor primary resistance. Treatment with 10 μM perifosine but not 50 μM DMF, enhanced the sensitivity of DLD-1 (*p* < 0.01) and HT-29 (*p* < 0.01) to the MEK inhibitors PD0325901 and trametinib (Figure 4A). In addition, co-treatment with perifosine and trametinib significantly elevated the Bax (*p* < 0.01) and Bim (*p* < 0.01) expression and reduced the expression of Bcl-2 (*p* < 0.01) and Bcl-xL (*p* < 0.01) (Figure 4B,C). These results indicate that overactivation of Akt in these two colorectal cancer cell lines is associated with MEK inhibitor primary resistance.

### 2.4. Establishment of MEK Inhibitor Resistance Colorectal Cancer Cells and Akt Overactivation Is Involved with MEK Inhibitor Resistance

**Figure 5:**
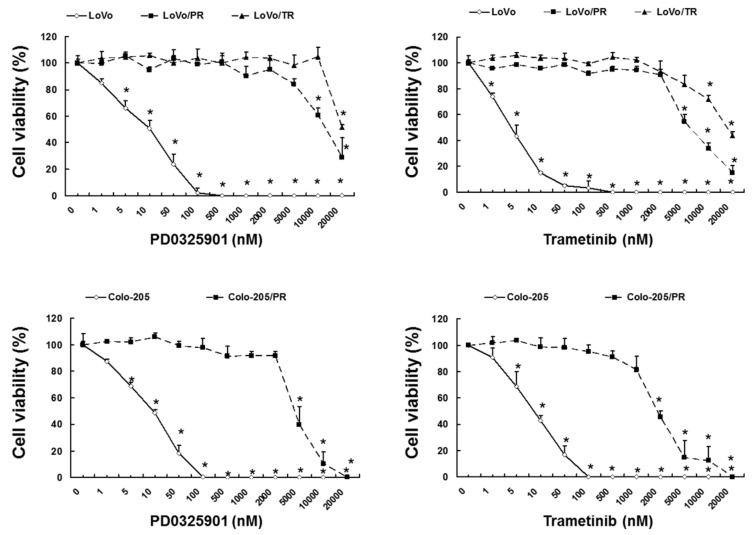
Viability of LoVo/PR, LoVo/TR or Colo-205/OR cells under trametinib or PD0325901 treatment. Cell viability of LoVo/PR, LoVo/TR or Colo-205/PR cells and their parental cell lines after administrated with indicated concentrations of PD0325901 and trametinib for 72 h. The results showed 5 independent experiments. * *p* < 0.05 vs. untreated LoVo or Colo-205 cells as assessed with Shapiro-Wilk test and Kruskal-Wallis test followed by Steel test.

**Figure 6:**
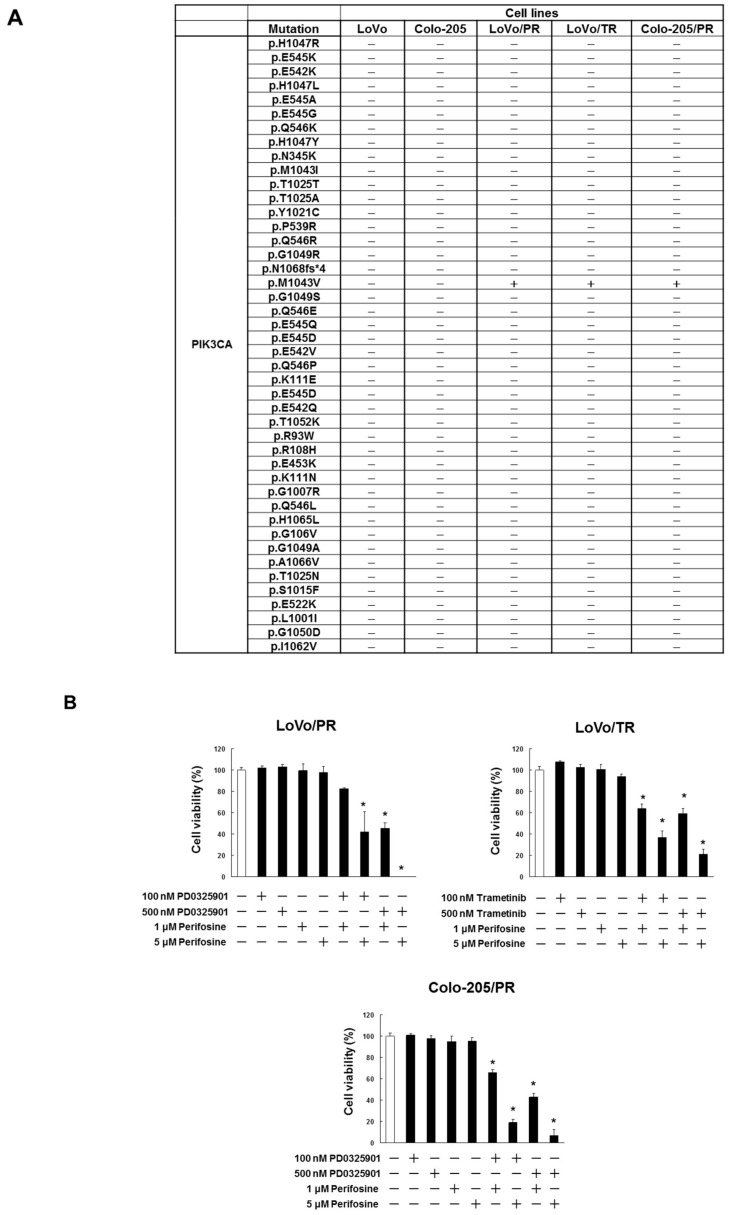

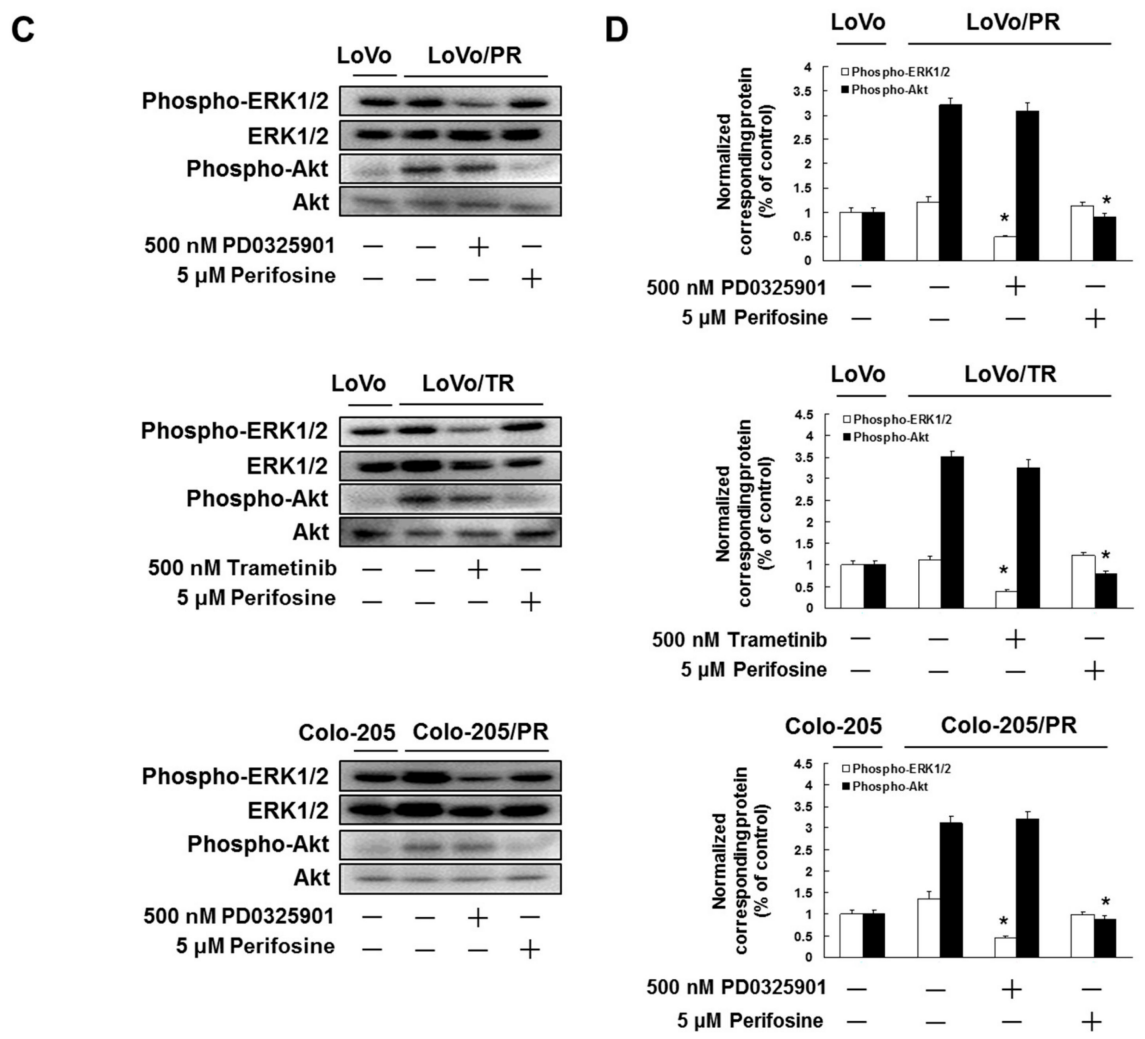
Detection of PIK3CA gene mutation in LoVo/PR, LoVo/TR or Colo-205/PR cells. (**A**) Genomic mutation analysis of PIK3CA using the Somatic Mutation polymerase chain reaction (PCR) array. (**B**) LoVo/PR, LoVo/TR or Colo-205/PR cells were administrated with indicated concentrations of trametinib, PD0325901 or perifosine. After incubation for 72 h, cell viability was analyzed by trypan blue staining. The results showed 5 independent experiments. * *p* < 0.05 vs. untreated cells as assessed with Shapiro-Wilk test and Kruskal-Wallis test followed by Steel test. (**C**) Cell lysates were examined by western blotting assay using indicated antibodies. (**D**) Quantification of protein expression, normalized corresponding protein, respectively. The results showed the 5 independent experiments. * *p* < 0.01 vs. LoVo/PR, LoVo/TR or Colo-205/PR untreated cells (Shapiro-Wilk test and ANOVA with Dunnet’s test).

To investigate whether acquired resistance to MEK inhibitor is associated with overactivation of Akt, we established the PD0325901- and trametinib-resistant LoVo/PR, Colo-205/PR or LoVo/TR sublines. We first confirmed the MEK inhibitor resistance of these sublines by determining their IC50 for PD0325901 and trametinib compared to parental LoVo or Colo-205 cells. We found that LoVo/PR, Colo-205/PR and LoVo/TR cells displayed a significantly higher IC50 for PD0325901 and trametinib than their respective parental LoVo or Colo-205 cell lines (IC_50_ value for trametinib and PD0325901: LoVo vs. LoVo/PR or LoVo/TR, *p* < 0.05, Colo-205 vs. Colo-205/PR, *p* < 0.05) (Figure 5 and Supplementary Materials, Figure S5). Next, we investigated the mutation status of the PIK3CA gene, which revealed that LoVo/PR, Colo-205/PR and LoVo/TR cells harbored an M1043V mutation (Figure 6A). Mutation of codon 1043 in PIK3CA is a “hot spot mutation” known to activate Akt signaling [24]. Importantly, we found that perifosine overcame the acquired resistance of LoVo/PR, Colo-205/PR and LoVo/TR cells to the MEK inhibitors PD0325901 and trametinib via inhibition of Akt activation (cell survival rate: *p* < 0.05, Akt phosphorylation: *p* < 0.01) (Figure 6B–D). These results demonstrate that overactivation of Akt contribute to MEK inhibitor acquired resistance and that Akt inhibitor can restore the sensitivity of acquired-resistant cells to MEK inhibitor.

## 3. Discussion

In the present study, we demonstrated that PIK3CA mutation contributes to MEK inhibitor primary resistance and that Akt is activated in response to MEK inhibitor treatment in the PIK3CA-mutated colorectal cancer cell lines DLD-1 and HT-29. Furthermore, a combination of MEK inhibitor and perifosine overcame the MEK inhibitor primary resistance in these colorectal cancer cell lines. DLD-1 and HT-29 cells harbor mutations in PIK3CA (E545K and P449T, respectively) which activate the kinase domain of PI3K and its downstream molecules, including Akt [25,26]. PIK3CA mutations (exon 9 and exon 20) and upregulation of Akt phosphorylation are associated with significantly decreased survival for colorectal cancer patients undergoing chemotherapy [27,28,29]. In addition, PIK3CA mutations are significantly associated with resistance to the anti-EGFR treatments panitumumab and cetuximab, as well as progression free survival in colorectal cancer patients [30]. It was reported that the treatment of refractory colorectal cancer with regorafenib, an inhibitor of multiple kinases, including vascular endothelial growth factor receptor (VEGFR) 1–3, Tie2, KIT, platelet derived growth factor receptor and RET, achieved a 6% partial response and 23% stable disease. However, poor progression free survival was observed in patients harboring PIK3CA mutations (PI3K upregulation) in this study [30]. It was also demonstrated that treatment with selumetinib failed to induce cell death and caspase-3/7 activity in HCT-116 cells harboring KRAS (G13D) and PIK3CA (H1047R) mutations [31]. In addition, selumetinib did not induce cell death in HCT-15 cells harboring KRAS (G13D) and PIK3CA (E545K) mutations [32]. Moreover, trametinib or selumatinib did not induce apoptosis in RKO cells harboring mutations in BRAF (V600E) and PIK3CA (P449T) [33]. Activation of the PI3K/Akt pathway has been implicated in resistance to both MEK inhibitor treatment in gastric cancer cells and BRAF/MEK inhibitor combination treatment in melanoma cells [34,35]. A multicenter phase II study of the MEK inhibitor CI-1040 revealed insufficient antitumor activity in patients with various cancers, including colorectal cancer [36]. In addition, in a phase I study of the MEK inhibitor RO4987655, progressive disease was observed in patients with KRAS/PIK3CA-mutant colorectal cancer [18]. Taken together, these findings suggest that overactivation of Akt contributes to MEK inhibitor resistance in KRAS and BRAF mutant colorectal cancer.

ERK1/2 and Akt activation are known to regulate the expression of many apoptosis-controlling factors. Up-regulation of pro-apoptotic factors such as Bax and Bim and down-regulation of anti-apoptotic factors such as Bcl-2 and Bcl-xL leads to caspase activation and apoptosis [37,38,39,40,41]. In this study, MEK inhibitor suppressed Bcl-2 and Bcl-xL expression and enhanced Bax and Bim expression in MEK inhibitor-sensitive cells but did not affect expression of any of the proteins in MEK inhibitor-resistant cells. In addition, a combination of trametinib and perifosine significantly suppressed Bcl-2 and Bcl-xL expression and enhanced Bax and Bim expression in MEK inhibitor-resistant cells. It has been reported that peroxiredoxin-2 activates the PI3K/Akt pathway, reduces Bax expression and enhances the expression of Bcl-2, thereby contributing to 5-fluorouracil resistance [42]. In addition, the overexpression of peroxiredoxin-2 induces Akt activation in human colorectal cancer tissues [42]. It was also reported that activation of the EGFR/Akt pathway by inhibition of hepatocellular carcinoma-related protein-1 suppressed Bim expression in human colorectal cancer cells and contributed to metastasis and poor prognosis in colorectal cancer patients [43]. In addition, it has been reported that inhibition of the PI3K/Akt/mammalian target of rapamycin (mTOR) pathway by apigenin enhances Bax expression and suppresses Bcl-2 expression and tumor cell growth in cisplatin-resistant human colorectal cancer cells [44]. Another study demonstrated that Akt phosphorylation by lipopolysaccharide stimulates resistance to oxaliplatin and doxorubicin in human colorectal cancer cells via increased Bcl-xL expression [45]. These findings suggest that Akt activation by PIK3CA mutation may contribute to MEK inhibitor resistance via regulation of Bcl-2, Bcl-xL, Bax and Bim expression.

In this study, we found that LoVo/PR, LoVo/TR and Colo-205/PR cells were resistant to trametinib and PD0325901 and contained a PIK3CA (M1043V) mutation leading to Akt activation. In addition, perifosine overcame the MEK inhibitor resistance in LoVo/PR, LoVo/TR and Colo-205/PR cells. The M1043V mutation of PIK3CA is known to activate the PI3K p110α kinase subunit [46] and has been detected in colorectal cancer patients [47]. It has also been shown that PI3K/Akt/mTOR inhibitor treatments have minimal activity against advanced colorectal cancer with PIK3CA-mutations including E545K, M1043V and H1047R and that concomitant KRAS mutations frequently contribute to this resistance [47]. In addition, a phase I study of buparlisib (BKM120), a pan PI3K inhibitor, showed that the best response was observed as stable disease in six patients with colorectal cancer [48]. Moreover, combining perifosine with capecitabine in patients with metastatic colorectal cancer improved the median time to progression and overall survival [49]. These findings suggest that PIK3CA, KRAS or BRAF mutations may contribute to MEK or PI3K inhibitor resistance and that a combination of Akt and MEK inhibitors may be a useful pharmacotherapeutic approach for colorectal cancer harboring dual mutations of PIK3CA and KRAS or BRAF.

This study present few limitations. Although we conducted four human colorectal cancer cell lines in this experiments, did not confirm other human colorectal cancer cell lines harboring KRAS, BRAF and PIK3CA mutation and patients-derived MEK inhibitor resistance colorectal cancer cells. In addition, it is not clear yet that combined treatment with trametinib or PD0325901 and perifosine suppressed the tumor growth in xenograft mouse model of MEK inhibitor resistance primary and acquired colorectal cancer cells. Thus, future studies will examine the inhibition of tumor growth by co-treatment with perifosine and trametinib or PD0325901 in mouse xenograft models of DLD-1, HT-29, our establishment MEK inhibitor resistance cells and patient-derived colorectal cancer cells. Moreover, we will examine the enhanced the cell death by combined treatment with perifosine and trametinib or PD0325901 in other human colorectal cancer cell lines harboring KRAS, BRAF and PIK3CA mutation. Moreover, it has been reported that a phase 2 study of MK-2206, an Akt inhibitor and selumatinib showed no objective response in patients with colorectal cancer and these inhibitors did not significantly suppress the phosphorylation of ERK1/2 and Akt in tumor tissues [50]. This finding indicates that it is necessary to maintain plasma concentrations of Akt and MEK inhibitors to significantly suppress ERK1/2 and Akt activation in tumor tissues; therefore, we will investigate the in vivo pharmacokinetic and pharmacodynamics profiles of perifosine, trametinib or PD0325901 alone and their combined treatment.

## 4. Materials and Methods

### 4.1. Cell Culture

DLD-1 cells were purchased from the Health Science Research Resources Bank (Osaka, Japan). LoVo, Colo-205 and Caco-2 cells were purchased from Riken Cell Bank (Ibaraki, Japan). HT-29 cells were purchased from DS Pharma Biomedical (Osaka, Japan). These cells were cultured in RPMI1640 medium (Sigma, St Louis, MO, USA), HamF12 medium (Sigma) or McCoy’s 5A medium (Sigma) supplemented with 10% fetal bovine serum (Gibco, Carlsbad, CA, USA), 100 μg/mL penicillin (Gibco), 100 U/mL streptomycin (Gibco) and 25 mM 4-(2-hydroxyethyl)-1-piperazineethanesulfonic acid (pH 7.4; FUJIFLIM Wako, Tokyo, Japan) in an atmosphere containing 5% CO_2_.

LoVo and Colo-205 cells with acquired resistance to PD0325901 (LC Laboratories, Woburn, MA, USA) or trametinib (LC Laboratories) (LoVo/PR, Colo-205/PR and LoVo/TR) were generated as previously described [37,51,52,53,54,55].

### 4.2. Trypan Blue Dye Exclusion Assay

The effect of PD0325901, trametinib, perifosine (SelleckChem, Houston, TX, USA) or DMF (FUJIFILM Wako) on cell viability was examined using the trypan blue stain exclusion assay as previously described [37,51]. DLD-1, HT-29, LoVo, Colo-205, LoVo/PR, Colo-205/PR and LoVo/TR cells were seeded onto flat-bottom 96-well plates for 24 h. Next, DLD-1, HT-29, LoVo and Colo-205 cells were treated with various concentrations of PD0325901 or trametinib for 1, 3 or 5 days; combination treatment with PD0325901 or trametinib and perifosine or DMF for 3 days; and LoVo/PR, LoVo/TR and Colo-205/PR cells were treated with various concentrations of PD0325901 or trametinib with or without perifosine for 3 days. A 0.4% trypan blue solution was mixed with the cell cultures and loaded into a hemocytometer. The cell survival rate represented the survival ((unstained cells)/death (stained cells)) rate on each day.

### 4.3. Western Blotting

DLD-1, HT-29, LoVo, Colo-205, Caco-2, LoVo/PR, Colo-205/PR and LoVo/TR cells were cultured under various conditions. In particular, DLD-1, HT-29, LoVo, Colo-205 and Caco-2 cells were cultured with medium for 2 days (Figure 1B,C). DLD-1, HT-29, LoVo and Colo-205 cells were treated with various concentrations of PD0325901 and trametinib for 3 days (Figure 2 and Figure 3). HT-29 cells were treated with trametinib and perifosine for 3 days (Figure 4B,C). LoVo/PR, LoVo/TR and Colo-205/PR were treated with PD0325901, trametinib or perifosine for 3 days (Figure 6C,D). Cell lysates were extracted by the ProteoExtract Subcellular Proteome Extraction Kit (Calbiochem, San Diego, CA, USA). The lysates were divided by electrophoresis and transferred to polyvinylidene fluoride membranes (GE Healthcare, Buckinghamshire, UK). The membranes were reacted with following primary antibodies according to the manufacturer’s instruction: anti-phospho-p44/42 MAPK (ERK1/2) antibody, anti-p44/42 MAPK (ERK1/2) antibody, anti-phospho-Akt antibody, anti-Akt antibody, anti-phospho-STAT3 antibody, anti-STAT3 antibody, anti-phospho-p38MAPK antibody, anti-p38MAPK antibody, anti-phospho-NF-κB antibody, anti- NF-κB antibody (Cell Signaling Technology, Beverly, MA, USA); anti-Bax antibody, anti-Bim antibody, anti-Bcl-xL antibody, anti-Bcl-2 antibody (Santa Cruz Biotechnologies, CA, USA); and anti-β-actin antibody (Sigma). Next, the membranes were reacted with horseradish peroxidase-connected secondary antibodies (GE Healthcare). The membranes were visualized by Luminata Forte (Merck Millipore, Nottingham, UK). The quantities of reactive proteins were determined based on densitometric measurements using a CS analyzer (ATTO, Tokyo, Japan) and reactive proteins were normalized to the corresponding proteins.

### 4.4. Somatic Mutation PCR Array

DLD-1, HT-29, LoVo, Colo-205, Caco-2, LoVo/PR, Colo-205/PR and LoVo/TR cells were cultured for 2 days and then DNA was extracted by the Nucleo Spin Tissue kit (Takara Biomedical, Siga, Japan). Somatic mutation of KRAS, BRAF and PIK3CA was examined by the SABiosciences qBiomarker Somatic Mutation polymerase chain reaction (PCR) array (QIAGEN, Germantown, MD, USA) according to the manufacturer’s instructions.

### 4.5. Statistical Analysis

All results are represented as means and standard deviations (SDs) of several independent experiments. All analyses were conducted using SPSS version 21.0 software and Shapiro-Wilk analysis and one-way analysis of variance (ANOVA) were performed. When no differences on Shapiro-Wilk and satisfactory differences on ANOVA were confirmed, the control group and various drug-treated groups were compared and analyzed using Dunnet’s test. When our data is not distributed, these were analyzed using Kruskal-Wallis test followed by Steel test. *p* values less than 0.05 were deemed significant.

## 5. Conclusions

In summary, this study demonstrated that overactivation of Akt is associated with MEK inhibitor resistance in colorectal cancer cells harboring KRAS or BRAF mutations and that Akt inhibitor overcame MEK inhibitor resistance. These findings suggest that a combination of Akt and MEK inhibitors may be a useful strategy for the treatment of colorectal cancer harboring overactivation of Akt.

## Supplementary Materials

The following are available online at https://www.mdpi.com/2072-6694/11/12/1866/s1, Figure S1: Detection of KRAS gene mutations in Caco-2, DLD-1, HT-29, LoVo and Colo-205 cells, Figure S2: Detection of BRAF gene mutations in Caco-2, DLD-1, HT-29, LoVo and Colo-205 cells, Figure S3: Detection of PIK3CA gene mutations in Caco-2, DLD-1, HT-29, LoVo and Colo-205 cells, Figure S4: Effect of trametinib and PD0325901 on cell viability in DLD-1, HT-29, LoVo and Colo-205 cells, evaluated by Muse™ Count and Viability kit, Figure S5: IC50 of various MEK inhibitors for LoVo, LoVo/PR, LoVo/TR, Colo-205 and Colo205/PR cells.
