# Supplementary material for: Overactivation of Akt Contributes to MEK Inhibitor Primary and Acquired Resistance in Colorectal Cancer Cells
Source: Cancers (Basel). 2019 Nov 25;11(12):1866. doi: 10.3390/cancers11121866 (PMC6966459; doi:10.3390/cancers11121866)
Supplement: Supplementary file 1 [file cancers-11-01866-s001.pdf]

cancers

Supplementary Materials: Overactivation of Akt Contributes to MEK Inhibitor Primary and Acquired Resistance in Colorectal Cancer Cells

		Cell lines				
	Mutation	Caco-2	DLD-1	HT-29	LoVo	Colo-205
KRAS	p.G12D	–	–	–	–	–
	p.G12V	–	–	–	–	–
	p.G13D	–	–	–	–	–
	p.G12C	–	–	–	–	–
	p.G12A	–	–	–	–	–
	p.G12S	–	–	–	–	–
	p.G12R	–	–	–	–	–
	p.G13C	–	–	–	–	–
	p.Q61H	–	–	–	–	–
	p.G13S	–	–	–	–	–
	p.Q61L	–	–	–	–	–
	p.Q61H	–	–	–	–	–
	p.A146T	–	–	–	–	–
	p.G13R	–	–	–	–	–
	p.Q61R	–	–	–	–	–
	p.G12F	–	–	–	–	–
	p.G13A	–	–	–	–	–
	p.Q61K	–	–	–	–	–
	p.G13D	–	+	–	+	–
	p.G13V	–	–	–	–	–

Figure S1. mutation analysis of KRASusing the Somatic Mutation PCR arrayin Caco-2, DLD-1, HT-29, LoVo, and Colo-205 cells.

		Cell lines				
	Mutation	Caco-2	DLD-1	HT-29	LoVo	Colo-205
BRAF	p.V600E	–	–	+	–	+
	p.V600K	–	–	–	–	–
	p.K601E	–	–	–	–	–
	p.V600R	–	–	–	–	–
	p.V600M	–	–	–	–	–
	p.V600A	–	–	–	–	–
	p.V600E	–	–	–	–	–
	p.G469A	–	–	–	–	–
	p.V600D	–	–	–	–	–
	p.V600G	–	–	–	–	–
	p.G469V	–	–	–	–	–
	p.G466V	–	–	–	–	–
	p.L597V	–	–	–	–	–
	p.L597S	–	–	–	–	–
	p.L597Q	–	–	–	–	–
	p.L597R	–	–	–	–	–
	p.G464E	–	–	–	–	–
	p.G469E	–	–	–	–	–
	p.Y472S	–	–	–	–	–
	p.G464V	–	–	–	–	–

Figure S2. mutation analysis of BRAFusing the Somatic Mutation PCR arrayin Caco-2, DLD-1, HT-29, LoVo, and Colo-205 cells.

		Cell lines				
	Mutation	Caco-2	DLD-1	HT-29	LoVo	Colo-205
PIK3CA	p.H1047R	–	–	–	–	–
	p.E545K	–	+	–	–	–
	p.E542K	–	–	–	–	–
	p.H1047L	–	–	–	–	–
	p.E545A	–	–	–	–	–
	p.E545G	–	–	–	–	–
	p.Q546K	–	–	–	–	–
	p.H1047Y	–	–	–	–	–
	p.N345K	–	–	–	–	–
	p.M1043I	–	–	–	–	–
	p.T1025T	–	–	–	–	–
	p.T1025A	–	–	–	–	–
	p.Y1021C	–	–	–	–	–
	p.P539R	–	–	–	–	–
	p.Q546R	–	–	–	–	–
	p.G1049R	–	–	–	–	–
	p.N1068fs*4	–	–	–	–	–
	p.M1043V	–	–	–	–	–
	p.G1049S	–	–	–	–	–
	p.Q546E	–	–	–	–	–
	p.E545Q	–	–	–	–	–
	p.E545D	–	–	–	–	–
	p.E542V	–	–	–	–	–
	p.Q546P	–	–	–	–	–
	p.K111E	–	–	–	–	–
	p.E545D	–	–	–	–	–
	p.E542Q	–	–	–	–	–
	p.T1052K	–	–	–	–	–
	p.R93W	–	–	–	–	–
	p.R108H	–	–	–	–	–
	p.E453K	–	–	–	–	–
	p.K111N	–	–	–	–	–
	p.G1007R	–	–	–	–	–
	p.Q546L	–	–	–	–	–
	p.H1065L	–	–	–	–	–
	p.G106V	–	–	–	–	–
	p.G1049A	–	–	–	–	–
	p.A1066V	–	–	–	–	–
	p.T1025N	–	–	–	–	–
	p.S1015F	–	–	–	–	–
	p.E522K	–	–	–	–	–
	p.L1001I	–	–	–	–	–
	p.G1050D	–	–	–	–	–
	p.I1062V	–	–	–	–	–

HT-29: Previously reported p.P449Tmutations not covered by our assays.(Oncogenesis. 2013 Sep 16;2:e71.)

Figure S3. mutation analysis of PIK3CAusing the Somatic Mutation PCR arrayin Caco-2, DLD-1, HT-29, LoVo, and Colo-205 cells.

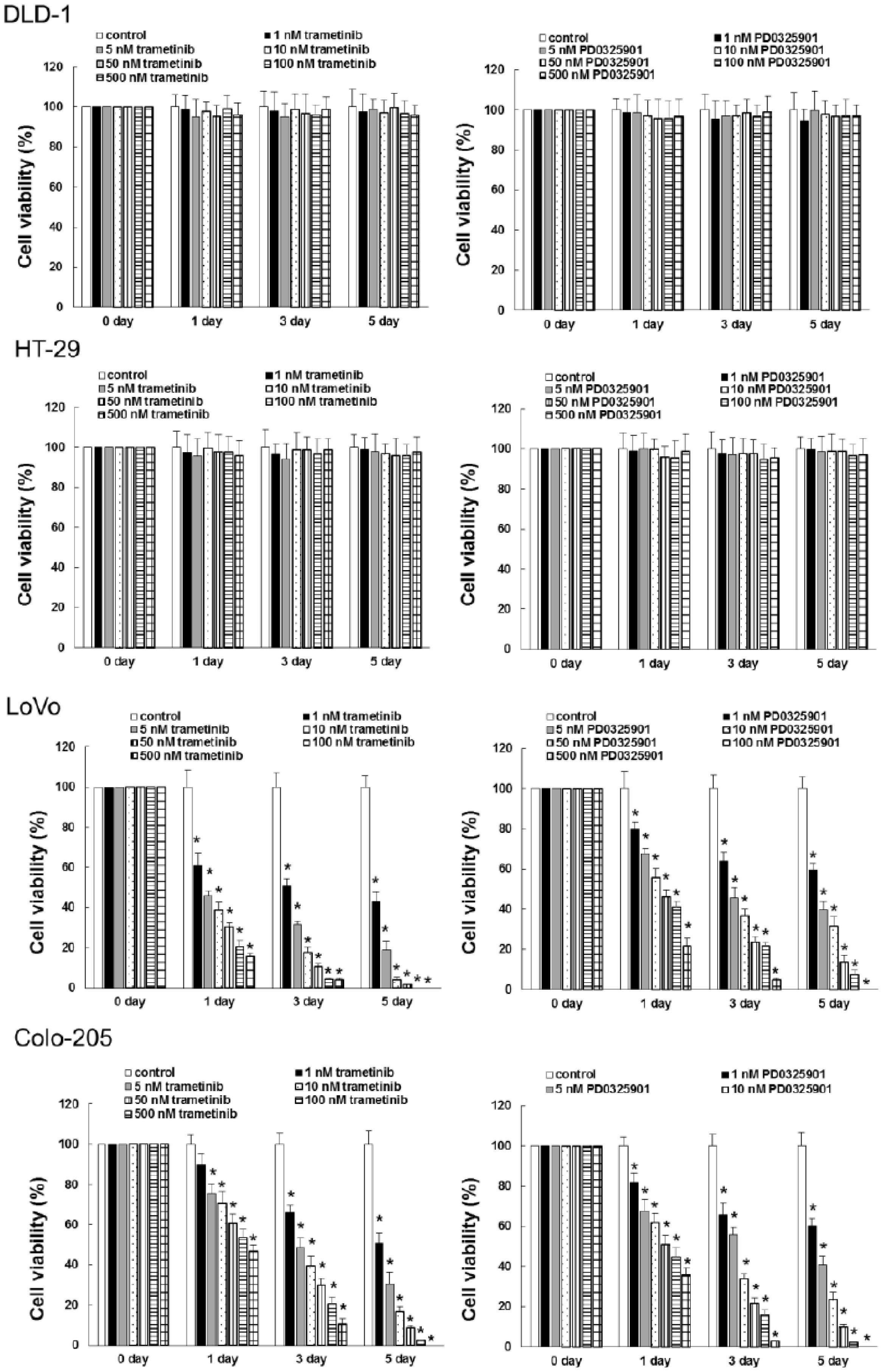
DLD-1
control
1 nM trametinib
5 nM trametinib
10 nM trametinib
50 nM trametinib
100 nM trametinib
500 nM trametinib
Cell viability (%)
0 day
1 day
3 day
5 day
control
1 nM PD0325901
5 nM PD0325901
10 nM PD0325901
50 nM PD0325901
100 nM PD0325901
500 nM PD0325901
HT-29
LoVo
Colo-205

Figure S4. of trametinib and PD0325901 on cell viability in DLD-1, HT-29, LoVo, and Colo-205 cells, evaluated by Muse™ Count and Viability kit.Cell viability of trametinib-and PD0325901-treated DLD-1, HT-29, LoVo, and Colo-205 cells as measured by the Muse™ Count and Viability kit(the dual DNA intercalating fluorescent dyes kit). These cells were administrated with indicated concentrations of trametinib and PD0325901 for 1, 3, or 5 days. The results showed the 5 independent experiments. *$p < 0.05$vs. controls (Shapiro-Wilk test and Kruskal-Wallis test followed by Steel test).

Cell line	IC_{50} (nM)	
	PD0325901	Trametinib
LoVo	11.507	3.404
LoVo/PR	10314.327	5949.128
IC_{50}-fold increase	896.352	1747.687
LoVo/TR	21522.609	19755.481
IC_{50}-fold increase	1870.392	5803.608
Colo-205	9.138	8.878
Colo-205/PR	4176.477	1846.866
IC_{50}-fold increase	457.045	208.027

Figure S5. Of various MEK inhibitors for LoVo, LoVo/PR, LoVo/TR, Colo-205, and Colo205/PRcells. Cell viability was analyzed by Trypan blue dye exclusion assay. The IC50s were calculated by fitting the data to a logistic curve.

Western Blots

Figure 1B

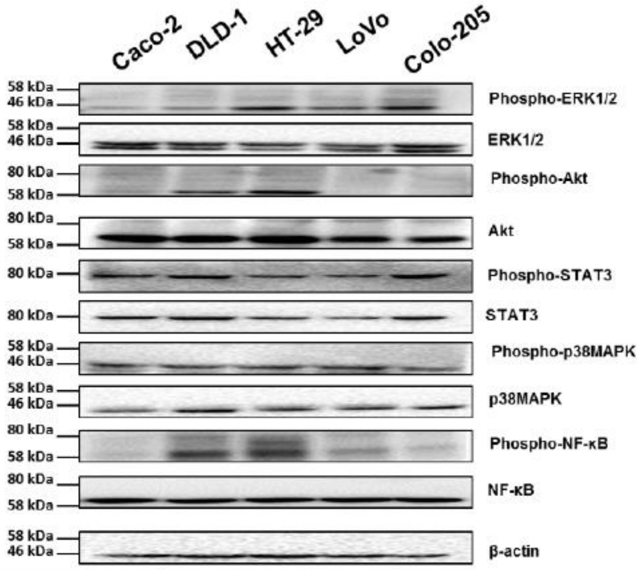

Phospho-ERK	1	3.212548	5.545456	4.687978	6.415447
ERK	1	1	1	1	1
Phospho-Akt	1	8.545469	12.45649	0.874878	0.798748
Akt	1	1	1	1	1
Phospho-STAT3	1	1.051314	0.879687	0.912135	1.121354
STAT3	1	1	1	1	1
Phospho-p38MAPK	1	0.854657	0.845468	0.869798	0.798469
p38MAPK	1	1	1	1	1
Phospho-NF-κB	1	10.12134	11.54655	1.123124	0.987988
NF-κB	1	1	1	1	1
β-actin	1	1	1	1	1

Figure 2

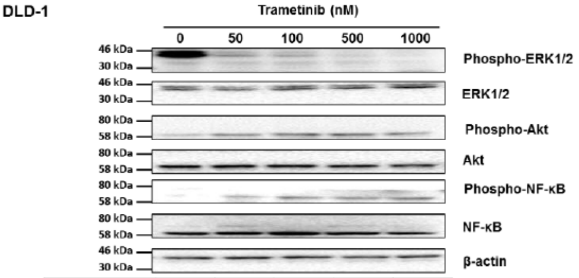

Phospho-ERK	1	0.251232	0.123155	0.087456	0.074565
ERK	1	1	1	1	1
Phospho-Akt	1	1.954665	2.451568	2.546469	2.646487
Akt	1	1	1	1	1
Phospho-NF-κB	1	1.546547	2.125465	2.546549	3.546469
NF-κB	1	1	1	1	1
β-actin	1	1	1	1	1

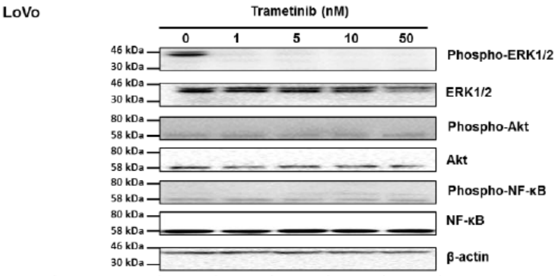

Phospho-ERK	1	0.212354	0.181353	0.074465	0.065548
ERK	1	1	1	1	1
Phospho-Akt	1	1.021314	0.954565	0.984547	1.051355
Akt	1	1	1	1	1
Phospho-NF-κB	1	0.984565	1.032135	1.084546	0.984655
NF-κB	1	1	1	1	1
β-actin	1	1	1	1	1

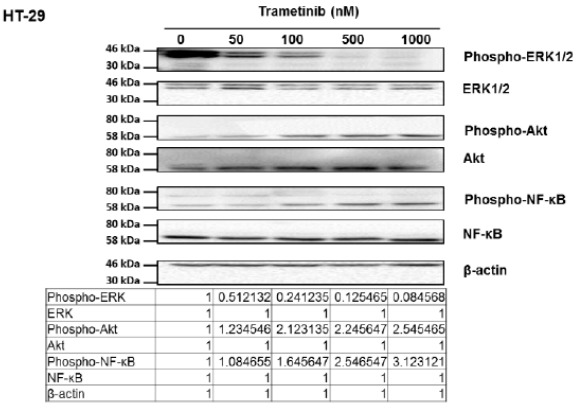

Phospho-ERK	1	0.512132	0.241235	0.125465	0.084568
ERK	1	1	1	1	1
Phospho-Akt	1	1.234546	2.123135	2.245647	2.545465
Akt	1	1	1	1	1
Phospho-NF-κB	1	1.084655	1.645647	2.546547	3.123121
NF-κB	1	1	1	1	1
β-actin	1	1	1	1	1

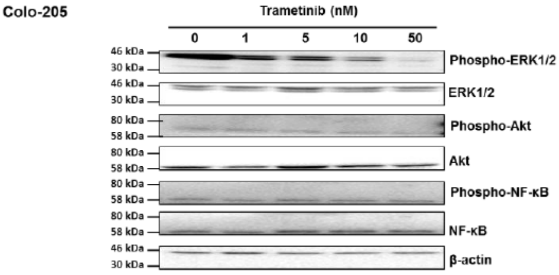

Phospho-ERK	1	0.546547	0.498465	0.312135	0.105465
ERK	1	1	1	1	1
Phospho-Akt	1	1.054562	1.064565	0.983455	0.954686
Akt	1	1	1	1	1
Phospho-NF-κB	1	1.054565	1.065467	1.081314	0.984547
NF-κB	1	1	1	1	1
β-actin	1	1	1	1	1

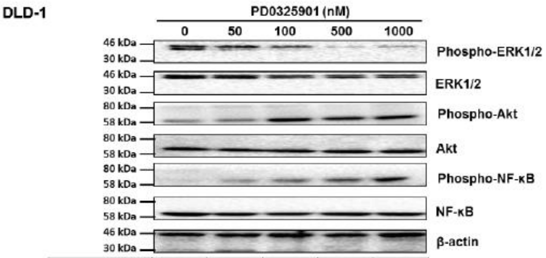

Phospho-ERK	1	0.821321	0.546868	0.498445	0.046879
ERK	1	1	1	1	1
Phospho-Akt	1	1.094546	2.412314	2.354655	2.513135
Akt	1	1	1	1	1
Phospho-NF-κB	1	1.254645	1.546549	1.846469	3.213155
NF-κB	1	1	1	1	1
β-actin	1	1	1	1	1

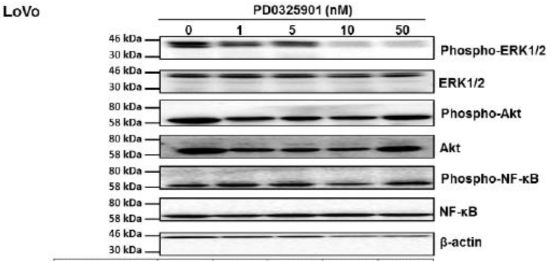

Phospho-ERK	1	0.856455	0.812152	0.235456	0.215645
ERK	1	1	1	1	1
Phospho-Akt	1	0.954547	0.945649	0.987879	1.012135
Akt	1	1	1	1	1
Phospho-NF-κB	1	0.965465	1.021321	0.974546	1.032132
NF-κB	1	1	1	1	1
β-actin	1	1	1	1	1

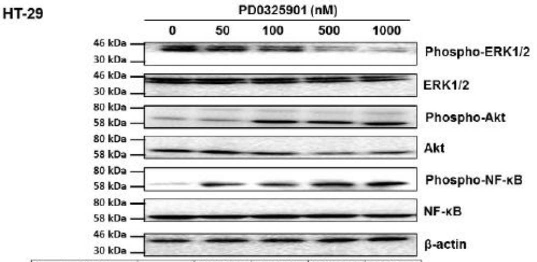

Phospho-ERK	1	0.813214	0.513214	0.213214	0.123135
ERK	1	1	1	1	1
Phospho-Akt	1	1.345469	2.513153	2.412314	2.846547
Akt	1	1	1	1	1
Phospho-NF-κB	1	1.946547	2.345647	2.413214	2.984649
NF-κB	1	1	1	1	1
β-actin	1	1	1	1	1

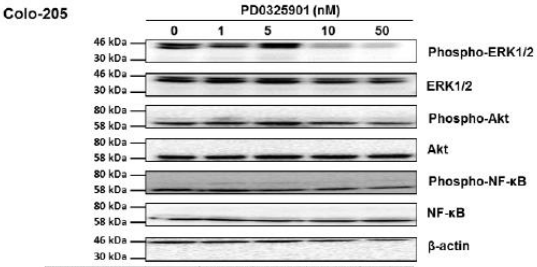

Phospho-ERK	1	0.894565	0.912135	0.345517	0.294565
ERK	1	1	1	1	1
Phospho-Akt	1	1.051324	0.984546	0.964566	0.965466
Akt	1	1	1	1	1
Phospho-NF-κB	1	1.032135	1.054546	0.984655	0.954688
NF-κB	1	1	1	1	1
β-actin	1	1	1	1	1

Figure 3

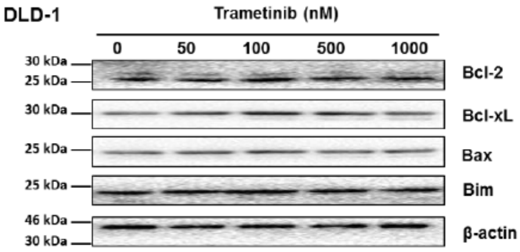

Bcl-2	1	0.945469	1.112132	1.021314	0.984655
Bcl-xL	1	1.151321	1.054655	1.032132	0.984655
Bax	1	1.032135	1.084546	0.976456	0.991321
Bim	1	0.954655	0.984655	1.021322	1.123122
β-actin	1	1	1	1	1

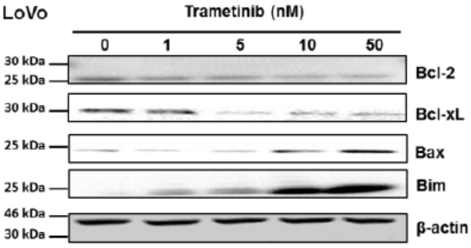

Bcl-2	1	0.745465	0.721315	0.512314	0.421312
Bcl-xL	1	0.545649	0.123135	0.184655	0.145647
Bax	1	1.054546	1.013153	2.121325	4.121354
Bim	1	2.454649	2.985467	4.454649	5.121131
β-actin	1	1	1	1	1

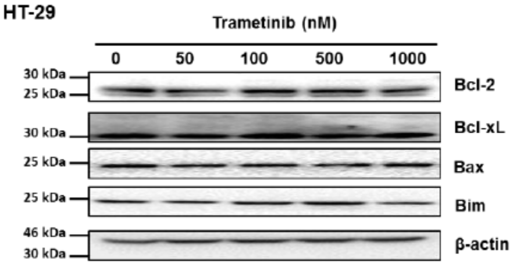

Bcl-2	1	1.021532	1.313213	1.313212	1.321355
Bcl-xL	1	0.984655	0.954547	1.102132	1.021321
Bax	1	0.984655	0.945465	1.054655	1.094547
Bim	1	0.955465	1.123114	1.081315	1.132135
β-actin	1	1	1	1	1

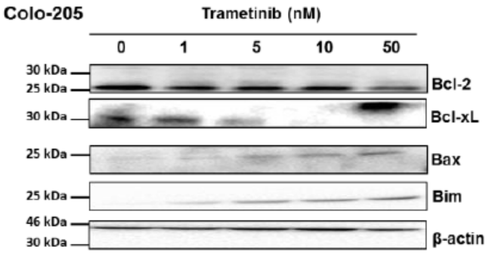

Bcl-2	1	0.845469	0.745477	0.721322	0.511315
Bcl-xL	1	1.121214	0.213215	0.185465	0.058688
Bax	1	0.984649	1.413544	2.213135	2.984649
Bim	1	1.345465	2.454647	2.946547	4.112135
β-actin	1	1	1	1	1

Figure 4B

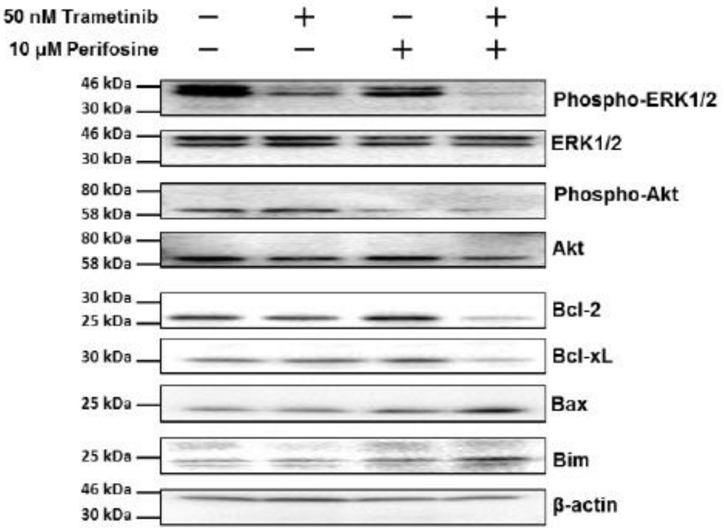

Phospho-ERK	1	0.405647	0.984547	0.231343
ERK	1	1	1	1
Phospho-Akt	1	0.894565	0.123155	0.105469
Akt	1	1	1	1
Bcl-2	1	0.894655	0.945647	0.423132
Bcl-xL	1	0.945649	0.813213	0.184565
Bax	1	1.051355	1.554658	4.213132
Bim	1	1.032313	1.456156	3.546568
β-actin	1	1	1	1

Figure 6C

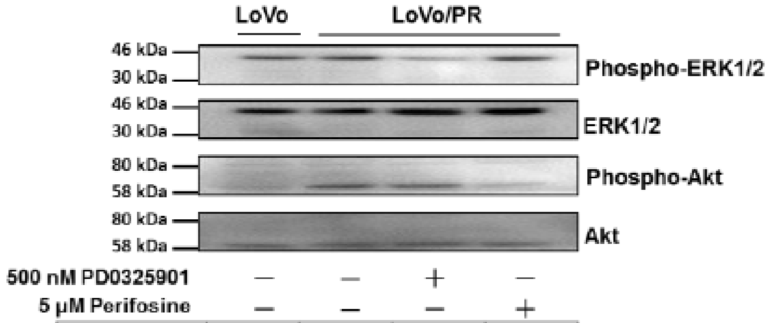

Phospho-ERK	1	1.213215	0.484565	1.121336
ERK	1	1	1	1
Phospho-Akt	1	3.213215	3.081314	0.891546
Akt	1	1	1	1

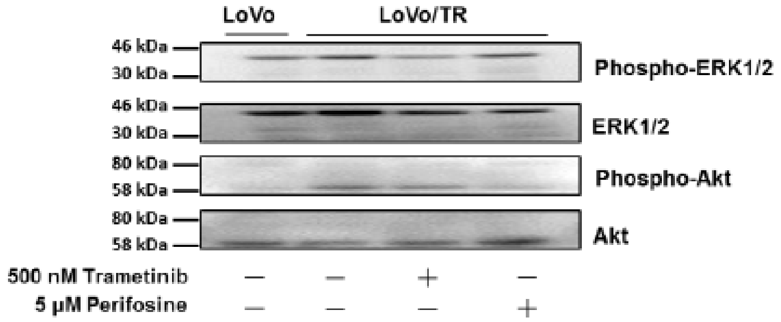

Phospho-ERK	1	1.123132	0.394565	1.213134
ERK	1	1	1	1
Phospho-Akt	1	3.513213	3.245467	0.794546
Akt	1	1	1	1

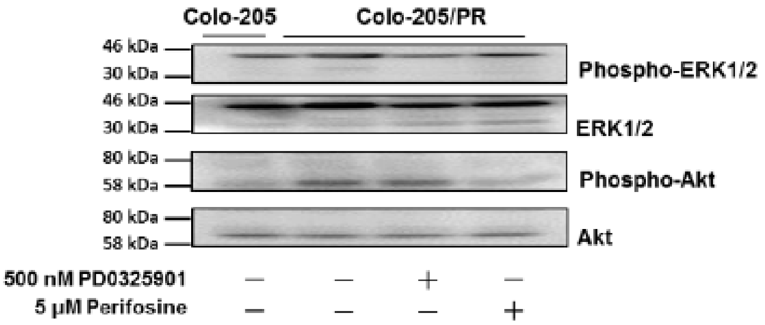

Phospho-ERK	1	1.345465	0.451321	0.984646
ERK	1	1	1	1
Phospho-Akt	1	3.123154	3.212315	0.884546
Akt	1	1	1	1

© 2019 by the authors. Submitted for possible open access publication under the terms and conditions of the Creative Commons Attribution (CC BY) license (http://creativecommons.org/licenses/by/4.0/).
